# Detection of Zoonotic *Photobacterium damselae* Subspecies *damselae* in a Razorbill ( *Alca torda* ): The First Report of a Potential Cross‐Species Transmission in Birds

**DOI:** 10.1111/1758-2229.70284

**Published:** 2026-02-12

**Authors:** Adriano Minichino, Francesca Lucibelli, Tullia Guardia, Rosario Balestrieri, Serena Aceto, Emanuela Vaccaro, Ludovico Dipineto, Marzia Sapio, Antonio Santaniello, Luigi Maria De Luca Bossa, Giovanna De Luca, Alessandro Fioretti, Luca Borrelli

**Affiliations:** ^1^ Department of Veterinary Medicine and Animal Productions University of Naples Federico II Napoli Italy; ^2^ Department of Biology University of Naples Federico II Napoli Italy; ^3^ Department of Integrative Marine Ecology Stazione Zoologica Anton Dohrn – CRIMAC, Calabria Marine Centre Amendolara Italy; ^4^ Regional Reference Center of Urban Veterinary Hygiene (CRIUV) Napoli Italy; ^5^ Istituto Zooprofilattico Sperimentale del Mezzogiorno Napoli Italy

**Keywords:** cross‐species transmission, new emerging zoonotic pathogen, *Photobacterium damselae* subsp. damselae, razorbill *(Alca torda)*, wildlife and seabirds disease

## Abstract

A razorbill ( *Alca torda* ) was found dead in Bacoli, Italy, on January 16, 2023, during an exceptional irruptive event. Post‐mortem examination revealed coelomitis with severe congestion of the liver, lungs, kidneys, and myocardium. Bacterial isolation, MALDI‐TOF MS analysis and PCR confirmed the presence of *Photobacterium damselae* subsp. *damselae* (Pdd), supported by 16S rDNA gene sequencing and detection of the *ureC* gene. PCR screening for virulence factors identified the *hlyAch* gene in lung samples, suggesting a potentially pathogenic strain in avian species. Histopathological examination showed severe inflammatory infiltrates and widespread haemorrhages with mild and multifocal lymphocytic infiltrates in tissues analysed. These findings suggest a significant role of Pdd in the observed lesions. Pdd is an emerging pathogen affecting a wide range of marine animals, including invertebrates, fish, and cetaceans. Here, we report the first isolation of Pdd in a razorbill and more importantly, the first detection in a migratory bird. We report a potential new cross‐species transmission of Pdd, underscoring its zoonotic potential and the need for further research. Given the role of migratory birds in pathogen spread and the impact of climate change on marine ecosystems, a preventive approach is essential to mitigate risks to wildlife, aquaculture, and human health.

## Graphical abstract

The first isolation of *Photobacterium damselae* subsp. *damselae* in a razorbill highlights a new cross‐species transmission of this new emerging zoonotic bacterium. Given the role of migratory birds in pathogen spread and the impact of climate change on marine ecosystems, further research and a preventive approach are essential to mitigate risks to wildlife, aquaculture, and human health.

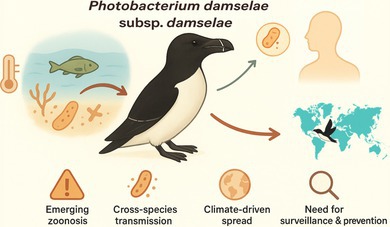

## 1 Introduction

*Photobacterium damselae* subsp. *damselae* is a marine bacterium with a global distribution, commonly found in various marine environments, including oceans, estuaries, seaweeds and sediments, and it thrives particularly well in coastal waters with temperatures between 20°C and 30°C (Alba et al. 2016; Osorio et al. 2018). It occurs both as a free‐living microorganism and as a pathogen associated with marine animals. It is considered an emerging pathogen affecting a wide range of hosts, including molluscs, crustaceans, fish, and cetaceans and is known to cause haemorrhagic septicemia in both wild and farmed populations, resulting in significant economic losses in the aquaculture industry (Ceccarelli and Colwell 2014; Gouife et al. 2022; Labella et al. 2017; Osorio et al. 2018; Rivas et al. 2013). Many recent studies report its isolation from newly cultured species and various geographical regions (Labella et al. 2017). However, the prevention and control strategies put in place are still limited (Gouife et al. 2022). In addition to its veterinary relevance, this microorganism is also a zoonotic pathogen capable of causing skin lesions in humans, which in severe cases may progress to necrotizing fasciitis with potentially fatal outcomes (Gouife et al. 2022; Rivas et al. 2013). Reported cases of *Photobacterium damselae* subsp. *damselae* infections in humans mainly originate from coastal areas of the United States, Australia, and Japan. Most infections result from wounds exposed to salt or brackish water, typically during fish handling or the use of fishing tools. Unusual cases of infection have also been reported following the ingestion of raw seafood and through the urinary tract due to exposure to seawater (Alvarez et al. 2006; Gouife et al. 2022; Rivas et al. 2013). Despite its notable ability to cross species barriers, to our knowledge, *Photobacterium damselae* subsp. *damselae* has not yet been reported in birds in the scientific literature.

The razorbill ( *Alca torda* , Linnaeus 1758) is an auk species breeding mainly in boreal and sub‐Arctic North Atlantic waters, with around 700,000 breeding pairs concentrated primarily in Iceland and the British Isles (Harrison et al. 2021). Following the breeding season, razorbills disperse to open waters of the North Atlantic where they typically overwinter (Lavers et al. 2007). Southward movements have also been observed, with individuals relocating along the Atlantic coasts of Portugal and France, and sporadically, as far as the Canary Islands and the western Mediterranean (de la Cruz et al. 2022; Lavers et al. 2007; Pasquet 1988). Occasionally, irruptive events may occur, resulting in unusually high numbers of specimens entering the Mediterranean, dispersing along its coastlines (Balestrieri et al. 2023; Boutabia et al. 2023). During the last mass irruption into the Mediterranean Sea in 2022/2023, razorbills showed peculiar feeding behaviours such as foraging close to shorelines or even within ports, as documented by photos and videos made by citizens (Balestrieri et al. 2023; Monti et al. 2024). Typically, their diet consists of energy‐rich clupeids such as capelin ( *Mallotus villosus* ), Atlantic herring ( *Clupea harengus* ), and sandeels (*Ammodytes* spp.), which sustain them through winter (Barrett 2015; Lavers et al. 2007). However, during the irruption, they adapted their feeding by targeting Mediterranean fish similar in size and shape to their usual prey (Monti et al. 2024).

Shifts in migration patterns or migratory ranges, as well as alterations in diet composition driven by changes in prey availability or environmental conditions, can increase the risk of exposure to unfamiliar pathogens, as altered stopover habitats or foraging niches are linked to variation in pathogen acquisition (Liao et al. 2024; Smith et al. 2023).

In this context, this study presents the first documented isolation of *Photobacterium damselae* subsp. *damselae* in a razorbill ( *Alca torda* ). This finding raises the possibility of a novel cross‐species transmission, highlighting the importance of continued surveillance due to the bacterium's known zoonotic potential.

## 2 Materials and Methods

**FIGURE 1:**
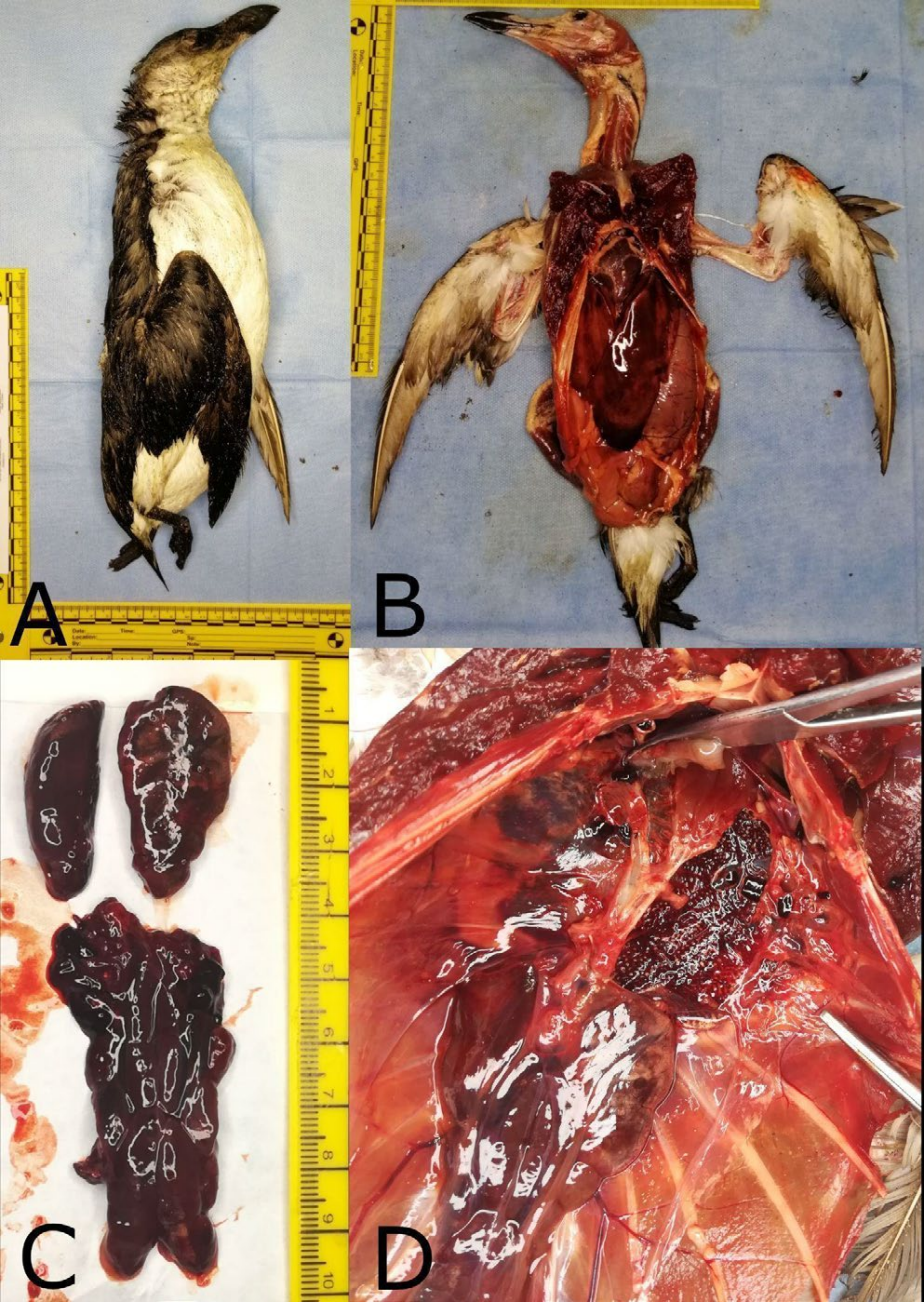
Gross pathologic examination of the Razorbill ( *Alca Torda* , Linnaeus 1758) (A); Appearance of coelomitis (B) secondary to septicemia with severe congestion of liver, kidneys (C), and lungs (D).

**FIGURE 2:**
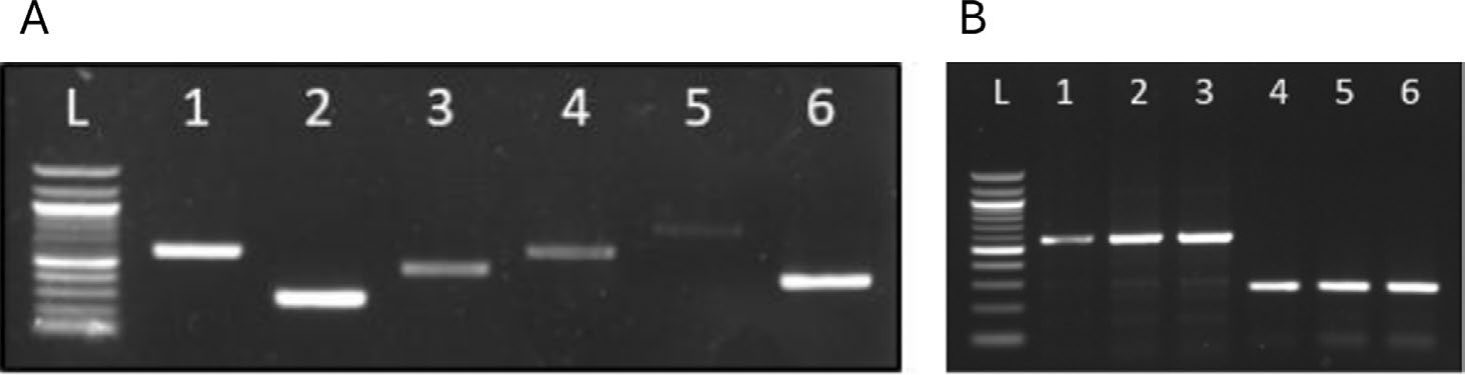
(A) Agarose gel electrophoresis of the amplification products of the *Photobacterium damselae* genes from lung tissue. 100 bp DNA Ladder (NEB, Ipswich, MA, USA) (L); 16S (1, 2); *ureC* (3); *dly* (4); *hlyA*_*pl*_ (5); *hlyA*_*ch*_ (6). (B) Agarose gel electrophoresis of the amplification products of *Photobacterium damselae* genes from liver, kidney and lung tissue. 100 bp DNA Ladder (NEB, Ipswich, MA, USA) (L); *16S* from liver (1, 4), kidney (2, 5) and lung tissue (lane 3, 6).

On January 16, 2023, a razorbill was found dead and beached in Bacoli (Naples), Italy, and subjected to a post‐mortem examination. The bird weighed 800 g and was identified as a young female, moderately emaciated. On gross examination, the cadaver exhibited mild autolysis and no external traumatic lesions. The coelomic cavity revealed non‐specific coelomitis with marked, diffuse congestion of the liver, lungs, kidneys, and myocardium (Figure 1). Death was attributed to septicemia, with cardiocirculatory arrest as the terminal event. Samples of the liver, lungs, kidneys, and heart were aseptically dissected using sterile techniques. Specimens of lung, kidney, liver, and heart were fixed in 10% buffered formalin, paraffin‐embedded, sectioned at 4 μm, and stained with haematoxylin and eosin (H&E). Lesions were evaluated in reference to the normal histological features of avian tissues. Sterile swabs were used for samples of lung, liver, spleen and kidney. Samples were cultured in thiosulfate citrate bile salts sucrose agar (TCBS) (Oxoid), on MacConkey Agar, and Tryptone Soy Agar containing 5% sheep blood (TSA), (Oxoid), and incubated at 37°C for 24 h. according to Morick et al. (Andreoni and Magnani 2014; Morick et al. 2023). Bacterial species were confirmed using a rapid, proteomic based technique for identification of clinical bacterial isolates by protein profiling using matrix‐assisted laser desorption ionisation‐time of flight mass spectrometry (MALDI‐TOF MS) systems. Molecular characterisation of bacterial species was conducted by PCR analysis. DNA was extracted from 1 g of dissected lung, liver/spleen and kidneys using the CTAB method (Doyle and Doyle 1999). PCR reactions were conducted to amplify regions of the 16S ribosomal DNA and *ureC* gene to assess the molecular attribution of *P. damselae*. The amplification of the *16S* rDNA gene is essential to demonstrate the positivity of *P. damselae* in the sample, while the *ureC* gene serves as a marker to distinguish between the subspecies of *P. damselae* as previously reported (Morick et al. 2023). To evaluate bacteria virulence, a PCR screening of haemolysin was conducted amplifying the pPHDD1 plasmid genes *damselysin* (*dly*) and *haemolysin A* (*hlyA*_*pl*_), and the chromosome gene *hlyA*_*ch*_ (Alba et al. 2016; Rivas et al. 2011). All PCR reactions were performed using the *DreamTaq* DNA polymerase (Invitrogen‐ThermoFisher, Waltham, MA, USA) based on the manufacturer's instructions. The nucleotide sequences of the primers used are listed in Table 1. Agarose gel (1.5%) electrophoresis was conducted on the amplification products at 100 V and the GelDoc Go Imaging System (Bio‐Rad, Hercules, CA, USA) was used for image acquisition. The amplification products of the 16S ribosomal DNA and *ureC* gene were directly sequenced by Sanger method (Eurofins Genomics, Ebersberg, Germany). PCR amplification using the primer pairs to amplify 16S, *ureC* and virulence genes produced fragments of the expected size (Figure 2, Table 1) (Morick et al. 2023; Rivas et al. 2014). The sequences were analysed using BLASTn and deposited in GenBank.

**TABLE 1:** Nucleotide sequence of the primers used.

Primer name	Sequence (5′–3′)	Target	Amplicon length (bp)
P1	TAGTGTAGTTAACACCTGCAC	16S	570
P2	ACACTCGAATCTCTTCAAGT		
CAR1	GCTTGAAGAGATTCGAGT	16S	267
CAR2	CACCTCGCGGTCTTGCTG		
URE5	TCCGGAATAGGTAAAGCGGG	*ureC*	448
URE3	CTTGAATATCCATCTCATCTGC		
DLYF	CCTATGGGACATGAATGG	*dly*	549
DLYR	GCTCTAGGCTAAATGAATC		
HLYA‐PLF	GCTATAAATGAATAAGAAAA	*hlyA*_*pl*_	767
HLYA‐PLR	TTGAAGCTAACTCAAAAA		
HLYA‐CHF	AATGTTTCTTTCCGTTGGGC	*hlyA*_*ch*_	353
HLYA‐CHR	CCGGAGTTCCACCAGTAAAT		

## 3 Results

**FIGURE 3:**
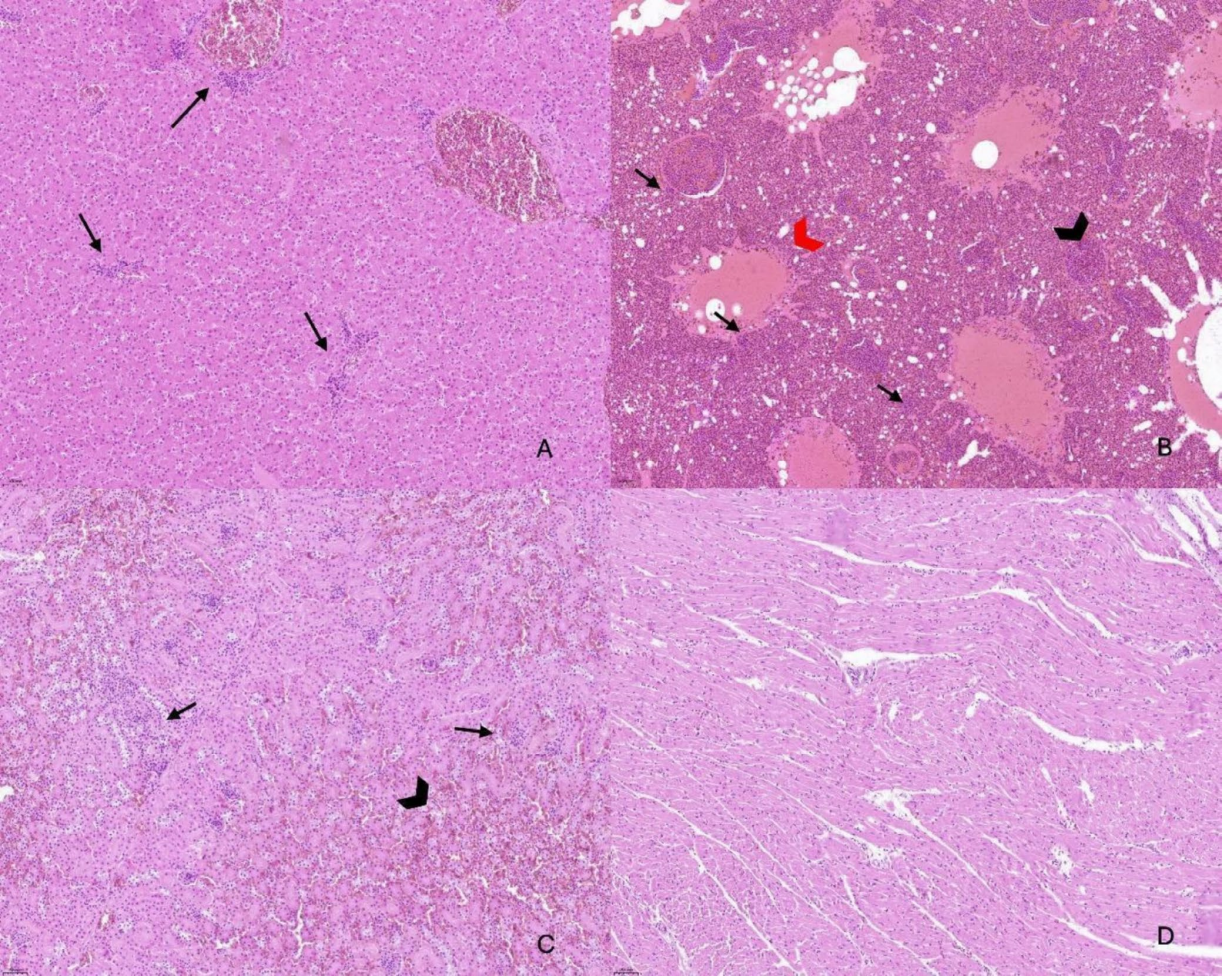
Histologic examination of the liver (A), lung (B), kidney (C), and heart (D) in Razorbill Bird. (A) The liver showed moderate, multifocal, and periportal infiltration of lymphocytes and plasma cells (arrows). (B) The lung showed capillary congestion (red arrowhead) and pulmonary edema (black arrowhead). Moderate infiltration of lymphocytes in the lung section was observed (arrows). (C) Numerous inflammatory cells (arrows) and diffuse haemorrhage (arrowhead) were observed in the kidney. (D) The heart showed few disseminated lymphocytes (arrows). Occasional neutrophils are also present within the inflammatory foci.

At necropsy, the cadaver showed mild autolysis without external traumatic lesions; non‐specific coelomitis was present with marked, diffuse congestion of the liver, lungs, kidneys, and myocardium, supporting septicemia as the underlying cause of death and cardiocirculatory arrest as the terminal event. Bacterial isolates from lung, liver, and kidney samples showed green colonies in TCBS agar, light‐grey colonies on MacConkey agar and beta haemolysis on TSA with 5% sheep blood consistent with the *Photobacterium* genus. *Photobacterium damselae* was the only species isolated from the razorbill samples that were also tested on selective and enriched media, which yielded no growth of other potentially pathogenic *Salmonella* spp., *Vibrio* spp., *Pseudomonas* spp., *Listeria* spp., and *Pseudomonas* spp. Identification of suspected colonies was performed via MALDI‐TOF MS with resulting log scores indicating a high‐confidence identification (log score > 2.0) and further characterised molecularly using PCR. Amplification and sequencing of the *16S* and *ureC* genes from DNA extracted from lung tissue confirmed the presence of *P. damselae* in that tissue. They allowed its attribution to the *P. damselae* subsp. *damselae* strain. The obtained sequences were deposited in GenBank under accession numbers PP946852 (16S), PP946853 (16S), and PP947936 (ureC). Furthermore, amplification of the *16S* gene from DNA extracted from liver and kidney tissues also revealed the presence of *P. damselae* in these tissues. PCR screening for virulence genes detected amplification of the *hemolysin* plasmid genes *damselysin* (*dly*) and *hlyApl*, as well as the chromosomal hemolysin gene *hlyAch*, in lung samples, suggesting that the isolated strain is potentially pathogenic (Labella et al. 2017; Matanza and Osorio 2020; Rivas et al. 2011). The amplification of the *haemolysin* genes shows that the *P. damselae* subsp. *damselae* strain found in the lung has virulence genes. Histopathological examination revealed inflammatory lesions affecting multiple organs. The liver showed marked perivascular inflammation, the lung exhibited congestion and edema associated with mild inflammation, and the kidney displayed extensive haemorrhages and tubular necrosis. The heart was only mildly affected, showing limited multifocal infiltration. Scattered neutrophils were also observed within the inflammatory foci, suggesting a subacute stage of bacterial infection. Detailed histological features are provided in the figure legends (Figure 3A–D).

## 4 Discussion

This study reports the first isolation of *Photobacterium damselae* subsp. *damselae* in a razorbill. Anatomopathological findings, which may be consistent with septicemia, together with microbiological and molecular results, suggest this is the first documented case of presumptive systemic infection by *P. damselae* subsp. *damselae* in this species. To our knowledge, this is also the first report of *P. damselae* subsp. *damselae* isolation in any bird. The isolated *Photobacterium damselae* strain was initially identified using MALDI‐TOF MS and subsequently confirmed by PCR amplification of the 16S rDNA gene. It exhibited growth at 37°C as well as hemolytic activity. These phenotypic traits differentiate *P. damselae* subsp. *damselae* from the subspecies *P. damselae* subsp. *piscicida* (Andreoni and Magnani 2014; Rivas et al. 2013). Subspecies identity was further confirmed by PCR targeting the *ureC* gene, a commonly used molecular marker for distinguishing between the two, as this gene is absent in the *P. damselae* subsp. *piscicida* genome (Morick et al. 2023). In addition, PCR analysis confirmed the presence of key virulence factor genes (*dly*, *hlyApl*, *hlyAch*) in lung samples, which are known to contribute to the pathogenicity of this subspecies. In particular, the pPHDD1 plasmid genes *damselysin* (*dly*) and *haemolysin A* (*hlyApl*) potentially contribute to *P. damselae* subsp. *damselae* ability to be pathogenic in marine habitats as well as for humans, as strains carrying pPHDD1 show markedly increased hemolytic activity compared to those encoding only the chromosomal *hlyAch* gene (Labella et al. 2017; Matanza and Osorio 2020; Rivas et al. 2011).

The finding of the razorbill specimen fits a context of irruption migration by seabirds observed along the coasts of the Mediterranean and the Tyrrhenian Seas during the winter of 2022/2023. This phenomenon has been attributed to extreme weather events in the North Atlantic, which may have contributed to the southward displacement of razorbills. Such displacement could have affected their physical condition, potentially due to challenges in locating or catching prey. Additionally, the suboptimal trophic conditions of Mediterranean waters for this species might have played a role (Balestrieri et al. 2023). These factors may have influenced the susceptibility of razorbills to infection by *P. damselae* subsp. *damselae*, as this microorganism is considered an opportunistic pathogen not only for humans, but also for a variety of marine animals, like cetaceans (Battistini et al. 2024). However, we cannot rule out that *P. damselae* subsp. *damselae* was already present when the razorbill arrived in the Mediterranean and that a stressful event associated with irruptive migration may have triggered the infection. It has also been demonstrated that warmer marine waters lead to the upregulation of virulence‐associated gene transcription, thereby enhancing its pathogenic potential and increasing the likelihood of infection (Matanza and Osorio 2020). Consequently, climate change is likely to impact not only the migratory and foraging patterns of razorbills and other seabirds (Diamond and Devlin 2003; Orgeret et al. 2022), but also to increase their exposure to potentially pathogenic agents such as *P. damselae* subsp. *damselae*. This isolation represents an important finding, as it expands the known host range of *P. damselae* subsp. *damselae* to include birds, highlighting its potential ecological significance. The razorbill was found just a few meters from the coast. Additionally, these birds feed on fish, including those consumed by humans, and have been observed scavenging fishery waste at ports as well as requiring food directly from people (Monti et al. 2024). Although the role of seabirds in spreading this pathogen remains hypothetical, migratory and sedentary birds are known to contribute significantly to the ecology and transmission of zoonoses, potentially serving as both mechanical and biological vectors (Georgopoulou and Tsiouris 2008; Navarro et al. 2019). Therefore, there is a potential risk that seabirds could contribute to the spread of this pathogen, even over long distances. Further investigation is needed to understand the possible implications of a potential dissemination of *P. damselae* subsp. *damselae* via seabirds, which could affect not only marine fauna but also fish farms, terrestrial animals and human health.

## 5 Conclusions

We reported the first isolation of *Photobacterium damselae* subsp. *damselae* in a razorbill. Our results, together with those in the literature, highlight how this emerging pathogenic bacterium could cross species barriers. Climate change and related extreme weather events and warming waters may facilitate the emergence and spread of pathogens like *P. damselae* subsp. *damselae*. Along with these changes, migratory birds can further contribute to the dissemination of infectious diseases even over long distances. This increases the risk of inducing disease in nearby and distant host populations with ecological and epidemiological implications for marine wildlife as well as aquaculture and human health. Consequently, mitigating this potential zoonotic risk necessitates an interdisciplinary coordinated action that integrates further research findings and the implementation of preventive measures.

## Author Contributions

**Adriano Minichino:** investigation, methodology, data curation, writing – original draft. **Francesca Lucibelli:** methodology, validation, formal analysis, writing – original draft. **Tullia Guardia:** writing – review and editing. **Rosario Balestrieri:** writing – review and editing. **Serena Aceto:** methodology, validation, formal analysis. **Emanuela Vaccaro:** methodology, visualisation. **Ludovico Dipineto:** writing – review and editing, visualisation, supervision. **Marzia Sapio:** methodology, writing – review and editing. **Antonio Santaniello:** visualisation. **Luigi Maria De Luca Bossa:** visualisation. **Giovanna De Luca:** visualisation, validation. **Alessandro Fioretti:** project administration, supervision. **Luca Borrelli:** supervision, investigation, methodology, data curation, writing – review and editing.

## Conflicts of Interest

The authors declare no conflicts of interest.

## Data Availability

All data generated or analysed during this study are included in this submitted article.
